# Commentary: A rapid action plan to improve diagnosis and management of lipodystrophy syndromes

**DOI:** 10.3389/fendo.2024.1445226

**Published:** 2024-09-10

**Authors:** William J. Massey, Lin Zhu

**Affiliations:** ^1^ Department of Inflammation and Immunity, Lerner Research Institute, Cleveland Clinic, Cleveland, OH, United States; ^2^ Center for Microbiome and Human Health, Lerner Research Institute, Cleveland Clinic, Cleveland, OH, United States; ^3^ Department of Medicine, Division of Diabetes, Endocrinology and Metabolism, Vanderbilt University Medical Center, Nashville, TN, United States

**Keywords:** lipodystrophy syndromes, adipose tissue, concomitant disease, rare endocrine disorders, metabolic disease, diabetic obesity

## 1 Introduction

Fourman et al. have recently published a clinically-oriented rapid action plan for the diagnosis and management of lipodystrophy syndromes (1). Lipodystrophy syndromes may be characterized by two factors: the pattern of fat loss which may occur across the whole body (generalized) or in specific body parts (partial) and whether the condition is genetic (congenital/familial) or acquired (2, 3). Unlike their clinical antithesis, obesity, lipodystrophy syndromes are rare, has heterogeneous clinical presentation, and are challenging to diagnose, yet the metabolic complications and end-organ damage that result from these two conditions can be similar. It took an average of 3.1 years in generalized lipodystrophy and 9.0 years in partial lipodystrophy for physicians to diagnose them accurately (4). Fourman et al. developed the quick action plan based on a large-scale study of comprehensive and long-term data across multiple countries (1, 4). An international panel of clinical experts were invited to discuss the key priorities for clinicians in the first 100 days after seeing a patient with clinical suspicion for the diagnosis (1). In comparison to the previous version of “step-by-step approach to diagnosis and treatment of lipodystrophy” (5), the rapid action plan has been updated with more detailed guidance for early diagnosis including the criteria for the skin thickness in men and women, which is considered important for early screening and can be precisely measured with dual-energy x-ray absorptiometry (1). Although the lack of adipose tissue is required for diagnosis of a lipodystrophy syndrome, common indicators that healthcare providers should watch for in their routine assessment of patients include features of metabolic syndrome such as insulin resistance, hypertriglyceridemia, fatty liver disease, and polycystic ovarian syndrome (PCOS) that is disproportionate to body size (i.e., in the absence of increased adiposity).

Treatment of lipodystrophies generally includes addressing the body’s inability to properly store lipid by limiting dietary fat, supplementation of adipokines, and common treatments for affected organs. The ultimate goal of the rapid action plan proposed by Fourman et al. is to improve the diagnosis for lipodystrophy syndromes to enable earlier and more consistent treatment for patients.

A prompt action in diagnosis and treatment for lipodystrophy can provide clinical data of the disease in its early stages, which is currently scarce due to its rarity and heterogeneity. Clinical data for lipodystrophy at its early stages are important to explore therapeutic strategies not only for lipodystrophy syndromes but also for diabetic obesity. The metabolic reflection of the lack of adipose tissue is similar in diabetic obesity, in line with the concept that the functional capacity of adipose tissue in diabetic obesity has been overwhelmed by nutrition. Lipodystrophies and diabetic obesity together highlight the critical roles of adipose tissue in metabolism. Research in lipodystrophy syndromes may provide critical information to explore novel therapeutic strategies for diabetic obesity. For example, metabolic disorders are reduced by leptin replacement therapy in generalized lipodystrophy patients (6, 7), which makes strategies of increasing signaling sensitivity to adipokines appealing to improve metabolic complications in diabetic obesity because adipokine levels are generally high in those patients.

## 2 Relevance to other concomitant diseases

While the Fourman et al. rapid action plan focuses on the clinical diagnosis and management of the metabolic consequences of lipodystrophy syndromes, it is important to note that other concomitant disease states may also contribute to lipodystrophy. These states include neurological diseases, autoimmune disorders, and inflammatory bowel diseases (IBD). Given the numerous insights lipodystrophy syndromes have provided in the metabolic disease space, further consideration should be given to lipodystrophies’ mechanistic relationships to other disease processes.

There have been reports of concomitant neurological diseases with lipodystrophy syndromes including a case report where a patient with congenital partial lipodystrophy and cataracts presented with paresthesia in the lower limbs and abnormal gait (8). Upon further investigation, the authors concluded that based on family history, the syndrome was likely autosomal dominant, but could not identify the underlying genetics. Subsequently, a study of six children carrying the c.985C>T mutation in the *BSCL2* (*Seipin*) gene lead to the association of the mutation with fatal neurodegeneration in homozygous carriers and this mutation was linked to exon 7-skipping transcripts of this gene (9). Most recently, a case series describing patients with a form of congenital generalized lipodystrophy presented with progressive myoclonus epilepsy was reported and attributed to novel mutations in the *BSCL2* (*Seipin*) gene (10). Importantly, none of these reports sought to understand the mechanistic basis of these neurological conditions with the lipodystrophy syndrome.

With respect to autoimmunity, it has been described that acquired generalized lipodystrophy can co-occur with type 1 diabetes. In a report of two such cases, the patients developed both conditions in prepubescence and exhibited poor glycemic control on insulin alone; however following a year of metreleptin treatment, both children had marked improvements in glycemic control with lower insulin requirements (7). Generalized lipodystrophy has also been acquired in pediatric patients with Hashimoto’s thyroiditis, rheumatoid arthritis, hemolytic anemia, and chronic hepatitis (6). In these cases, metreleptin therapy improved metabolic parameters, but did not affect the clinical course of the autoimmune conditions. The best described relationship between autoimmunity and lipodystrophies involves the C3 nephritic factor and acquired partial lipodystrophy. In these cases it was observed that autoimmune conditions such as systemic lupus erythematosus, dermatomyositis, and membranoproliferative glomerulonephritis consequently occurred with upper body partial lipodystrophy (11, 12). In three cases of chronic hepatitis with features of autoimmunity where patients developed generalized lipodystrophy, the mechanism of the observed low C4 complement factor levels was investigated. These studies showed that while the few patients had differing etiologies of autoimmunity constitutive activation of the alternative complement pathway appeared to be common between these cases (13). Collectively these reports indicate that both partial and general lipodystrophy syndromes do co-occur with numerous autoimmune conditions and therefore may influence one another.

There is a known relationship between adipose tissue and Crohn’s disease, one of the two main types of IBD. In Crohn’s disease there is an expansion of mesenteric adipose tissue that wraps around the diseased bowel wall, termed “creeping fat” (14, 15). Notably, there were two case reports in 2019 related to generalized lipodystrophy syndromes and Crohn’s disease. In the first case a pediatric patient with congenital generalized lipodystrophy experienced bowel perforation, which was associated with smooth muscle hypertrophy (16). The other report discussed a patient that experienced TNFα-dependent inflammation as a result of recombinant leptin therapy (17). Given the studies that have shown the contribution of adipose tissue to Crohn’s disease further work is warranted to clarify the molecular basis underpinning the role of lipodystrophy in Crohn’s disease pathology.

## 3 Conclusions

While Fourman et al. provide an up-to-date action plan for the clinical diagnosis and management of lipodystrophy syndromes’ numerous adverse metabolic consequences, there remains little work done in basic and translational research to understand the relationship between lipodystrophy syndromes and other concomitant conditions. Given the burden described in the studies cited above, especially in pediatric patients, and the tremendous insights that lipodystrophy syndromes have provided in the context of metabolic disease as well as adipose and lipid biology, greater consideration should be given to the role of adipose tissue cross talk with other organs involved in these concomitant conditions. Importantly, there are animal models available for lipodystrophies (18–20), which can facilitate such studies.
